# Tribute to Marcelle Grenson (1925–1996), A Pioneer in the Study of Amino Acid Transport in Yeast

**DOI:** 10.3390/ijms19041207

**Published:** 2018-04-16

**Authors:** Bruno André

**Affiliations:** Molecular Physiology of the Cell, Université Libre de Bruxelles (ULB), Biopark, 6041 Gosselies, Belgium; Bruno.Andre@ulb.ac.be

**Keywords:** yeast, amino acid transport, permease

## Abstract

The year 2016 marked the 20th anniversary of the death of Marcelle Grenson and the 50th anniversary of her first publication on yeast amino acid transport, the topic to which, as Professor at the Free University of Brussels (ULB), she devoted the major part of her scientific career. M. Grenson was the first scientist in Belgium to introduce and apply genetic analysis in yeast to dissect the molecular mechanisms that were underlying complex problems in biology. Today, M. Grenson is recognized for the pioneering character of her work on the diversity and regulation of amino acid transporters in yeast. The aim of this tribute is to review the major milestones of her forty years of scientific research that were conducted between 1950 and 1990.

## 1. First Steps at the Free University of Brussels

Marcelle Grenson was born on 25 June 1925 in Brussels (Schaerbeek). As she was gifted and diligent in school, her father (an electrician) wanted her to undertake secondary studies to become a teacher in primary school. In teacher training, she developed an interest in science. She was also interested in the logic of the electrical circuits her father designed at home, and she told me one day that those circuits may have triggered her early interest in regulatory mechanisms. After graduating, she earned enough as a teacher to help Andrée, her sister, through teacher training. Once Andrée was able to earn her living teaching, it was her turn to help her sister Marcelle to make a dream of many years come true: to study biology at the Free University of Brussels (*Université libre de Bruxelles*, ULB). In the final months of her undergraduate studies, M. Grenson did a master’s thesis under Prof. Raymond Jeener, who was head of the Animal Physiology Laboratory of the ULB, which was located in the vicinity of the *Rouge-Cloître*, near the Sonian Forest of Brussels. The subject of her master’s thesis was “Growth characteristics of a flagellate in an iron-free synthetic medium”. In 1950, she obtained her Master’s Degree in Zoological Science.


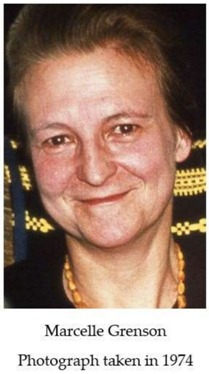


## 2. From the Role of RNAs in Protein Synthesis to Cytoplasmic Heredity

In 1950–1952, as Research Fellow of the Belgian Fund for Scientific Research (FNRS), M. Grenson undertook a doctorate focusing on the role of RNAs in protein synthesis. This topic was at the heart of the research conducted by R. Jeener and his colleague Jean Brachet, head of the Animal Morphology Laboratory of the ULB, and who had joined his colleague within the laboratories of the *Rouge-Cloître* shortly before the start of World War II. J. Brachet was among the first researchers to propose that RNA provided an information-containing matrix for protein synthesis [1,2]. During her doctorate, M. Grenson compared different biological systems (regenerating pigeon feather buds, chicken oviduct, mouse pancreas), and demonstrated that high protein synthesis activity is not necessarily associated with a high level of RNA synthesis (as measured by incorporation of radioactive phosphorus into RNA) [3,4,5,6]. This finding supported the notion that RNA acts as a template in protein synthesis, rather than playing a metabolic role (as donor of energy or phosphorus) or being merely a by-product or waste product of protein synthesis. In 1952, at the age of 27, she obtained the title of Doctor in Zoological Science. The same year she became an assistant in the Animal Physiology Laboratory of the ULB. Around the same time, she married René De Deken, who was a young researcher working in the Microbiology Laboratory of the ULB, which was headed by Prof. Jean-Marie Wiame.

In 1954, M. Grenson published a paper revealing major protein synthesis activity in the chloroplasts of etiolated chicory leaves, after their re-exposure to light [7]. This research involved the analysis of chloroplasts by electron microscopy, with equipment made available by Prof. J.-M. Wiame. This is probably the study that led M. Grenson to re-orient her work towards another topic: cytoplasmic heredity, and particularly towards a mysterious phenomenon: the irreversible loss of chloroplasts in cells treated with streptomycin. At the time, it was already well established that the properties of chloroplasts are determined not only by nuclear genes, but also by cytoplasmic entities that were endowed with genetic continuity, whose nature (DNA or RNA) and even location (in the cytoplasm or in the chloroplasts themselves?) remained unknown. M. Grenson conducted her first experiments on this subject on plants (barley, chicory, tobacco) [7], and later, from 1957, onward, on *Euglena*, which is an organism with which she had already worked during her master’s thesis. She thus had the opportunity to appreciate the advantages of working with *Euglena*: it can be grown in pure culture on natural or synthetic, liquid, or solid media whose nutrient composition can be varied. What’s more, it can live as a prototroph in the light and as a heterotroph in the dark. Lastly, it is possible to isolate mutants after various mutagenic treatments, such as exposure to X-rays or UV light. One notable achievement of M. Grenson in the study of cytoplasmic heredity was the rigorous demonstration that in *Euglena gracilis*, streptomycin and other treatments (heat shock, UV) cause a selective drop in the chloroplast multiplication rate, leading to a dilution of chloroplasts through successive cell divisions, and finally to their irreversible loss [8,9]. 

## 3. From *Euglena* to Yeast

In June 1959, M. Grenson was invited to present a seminar in Gif-sur-Yvette (France), in the Physiological Genetics Laboratory of Prof. Boris Ephrussi. The subject was “Hereditary loss of a biological function in Euglena: the synthesis of chlorophylls”. B. Ephrussi is considered to be the father of yeast genetics in France. With his student Piotr Slonimski, B. Ephrussi discovered in 1949 the famous “petite” mutants of yeast, which was obtained after treatment with acriflavin, an antiseptic used also as a dye. The phenotype of some of these respiration-deficient mutants is transmitted according to the principles of cytoplasmic heredity, and this suggested the existence of cytoplasmic particles that were endowed with genetic continuity. Some of the researchers interested in these cytoplasmic particles suspected that they consisted of RNA, already known to be abundant in the cytoplasm. It seems that M. Grenson initially shared this view, hypothesizing that streptomycin might target the same type of cytoplasmic particles in plants and *Euglena*, until she demonstrated that the antibiotic mainly targets chloroplast multiplication. This visit to Gif-sur-Yvette had a determining influence on future works of M. Grenson and her husband: the following year (1960), R. H. De Deken published a study on the appearance of “petite” mutants in different yeast species [10]. Its aim was to determine whether the appearance of these mutations correlated with sensitivity to euflavin, which is a compound that inhibits the respiratory system. As for M. Grenson, she most importantly realized how powerful yeast genetic methods are in tackling questions related to cell biology. In 1960, she submitted a project proposal (I found in her archives), wherein she proposed to develop in *Euglena* genetic methods that were similar to those that were developed for yeast. The difficulties encountered, however, led her quickly to give up her studies on *Euglena*, to focus entirely on yeast, and to become familiar with the genetic methods developed for the latter microorganism. A first step was to isolate, from a YF (“yeast foam”) yeast exposed to X-rays or UV radiation, tens of auxotrophic mutants. The first mutant isolated, strain D1, was a uracil auxotroph. M. Grenson then isolated “petite” mutants deficient in cellular respiration, which she classified according to whether transmission of the phenotype was Mendelian or cytoplasmic. Finally, as she had done previously with *Euglena*, she studied the effect of streptomycin on the frequency of appearance of these “petite” mutants. In January 1963, a chemistry student named Claire Squilbin, who was a judo enthusiast, joined M. Grenson’s team within R. Jeener’s laboratory. Her research focused on the influence of the yeast growth rate on the frequency of appearance of “petite” mutants. C. Squilbin was the last of M. Grenson’s students to study cytoplasmic heredity. M. Grenson gradually lost interest in this topic, particularly when it was discovered that both chloroplasts and mitochondria contain DNA. This discredited the notion, which was envisaged by M. Grenson and other researchers, that a molecule other than DNA, such as RNA, might be the genetic material determining certain properties of these cell organelles.

## 4. From Cytoplasmic Heredity to Amino Acid Transport

Another reason that led M. Grenson to abandon her work on cytoplasmic heredity was her growing interest in another process: amino acid transport. This subject was inspired by her increasingly regular exchanges with Jean-Marie Wiame, who was the head of the laboratory where R. H. de Deken was working, who also followed with great interest her experiments in yeast genetics. Given the importance of the long-term collaboration that has gradually been established between M. Grenson and J.-M. Wiame, I summarize below the early stages of Wiame’s scientific career. Then, I describe how this collaboration started. 

J.-M. Wiame (1914–2000), who was a chemist by training, completed his doctoral thesis under Professor Jean Brachet. At the time, J.-M. Wiame joined his laboratory, J. Brachet was already interested in protein synthesis and in the cytoplasmic substances that intervene in this process. As DNA is rich in phosphate, J. Brachet suggested to J.-M. Wiame that he analyze the possible role of the phosphate-rich cytoplasmic bodies, called volutin granules, in protein synthesis. J.-M. Wiame also taught microbiology at the *Institut Meurice*, a Brussels-based technical college having close ties with the National Institute of Fermentation Industries (INIF). In July of 1942, however, the German army closed down the ULB and its laboratories, including those that are located at the *Rouge-Cloître* site. J.-M. Wiame then decided to continue his experiments at the INIF. He was thus able to show that the phosphate-rich substance that was present outside the nucleus consists of polyphosphates [11], which are well known today to principally concentrate in the vacuole. J.-M. Wiame then did several postdoctoral research internships abroad. He worked first in St Louis, in the laboratories of C.F. Cori and G.T. Cori, then in New York, in those of Luigi Gorini and Werner Maas, on arginine metabolism in *E. coli*, and then in Berkeley, in that of Michael Doudoroff, on carbon catabolism in *Pseudomonas*. Upon returning to Belgium, he was assigned the Chair of Microbiology at the ULB and created, in 1948, a research institute associating the INIF with his Microbiology Laboratory of the ULB. This research institute (like the *Institut Meurice*) settled in 1955 on the campus of the *Centre d’Enseignement et de Recherche des Industries Alimentaires* (CERIA) in Anderlecht, where it became the *Institut de Recherches du CERIA* (IRC). The work of J.-M. Wiame in the 50s mainly concerned carbon and nitrogen metabolism in *Bacillus subtilis*, but also in other bacterial species and yeast. Later, initially working with R. H. Deken, he concentrated on arginine metabolism in yeast. For J.-M. Wiame, this amino acid was interesting because yeast cells can both synthesize and degrade it and could thus be expected to have a complex regulation ensuring the coordination of these two processes. J.-M. Wiame and coworkers began to explore this topic in earnest in the early 60s, which was a time when the metabolic regulation was primarily studied in bacteria. One of the leaders in bacterial metabolism was Jacques Monod, who J.-M. Wiame knew well and whose pioneering research on allostery and on gene regulation in *E. coli* likely had a great influence on J.-M. Wiame’s decision to reorient his research, from the biochemistry of diverse organisms to the regulation of metabolism in yeast. An anecdote illustrates well the closeness of these two scientists: during the events of May 1968, when Paris experienced a major shortage of fuel, J. Monod got in touch with J.-M. Wiame, asking him to bring jerry cans of gasoline to the Franco-Belgian border. 


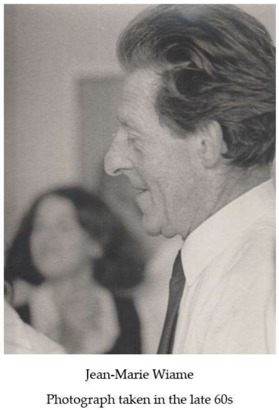


J.-M. Wiame’s interest in the first genetic experiments of M. Grenson’s team on yeast increased still further when, on one of his birthdays, she offered him three yeast mutants, strains AG1, AG2, and AG3, deficient in enzymes of arginine catabolism. This gift of strains, which added to the set of arginine auxotrophs (e.g., strain D20) that M. Grenson had already provided, marked the start of a long-term collaboration between M. Grenson and J.-M. Wiame. This collaboration initially focused on an unexplored question: how does yeast take up arginine from the external medium? There were several reasons for studying this question. In the early 60s, especially after publication of the pioneering work of G. Cohen, H. Rickenberg, G. Buttin, and J. Monod on the inducible β-galactoside permease (*lacY*) of *E. coli* [12,13], an increasing number of metabolite transport studies were being conducted on bacteria. Like the earlier work of M. Doudoroff’s laboratory on glucose transport in *E. coli*, the studies on the *lacY* permease that were carried out at the Pasteur institute in Paris had convincingly illustrated the power of genetics to reveal proteins that were specialized in transporting molecules. M. Grenson, with her mastery of the techniques used in yeast genetics, was well equipped to pursue a possible arginine permease in yeast. The timing was right, since in 1958, Harlyn Halvorson and Georges Cohen had shown it was possible to measure amino acid uptake activities in this organism [14]. M. Grenson became even more interested in arginine uptake when she and her husband observed, unexpectedly, that yeast strains auxotrophic for arginine or lysine were inhibited by streptomycin, an antibiotic to which most yeasts, including *S. cerevisiae*, are resistant. The explanation proposed by J.-M. Wiame was that streptomycin, which bears two guanidine groups, might interfere competitively with arginine uptake, assumed to be catalyzed by a permease. This is the context, marked by increasingly regular exchanges with J.-M. Wiame, in which M. Grenson isolated, between late 1961 and early 1962, in her small quarters at the *Rouge-Cloître*, the arginine transport defective mutant D58, the very first permease mutant in yeast. The affected permease was the arginine-specific permease Can1. The method used to isolate this mutant was that applied by Horowitz and Srb, in 1948, to the fungus *Neurospora crassa* [15] and by Schwartz, Maas, and Simon, in 1959, to bacteria [16]. It involves the isolating strains having become resistant to canavanine, which is a toxic arginine analog. Around the same time, in early 1962, J.-M. Wiame invited Madeleine Mousset, a young biologist interested in microbiology, to join his team for her master’s thesis. The aim of the proposed research was to study the uptake of radioactive arginine in wild-type yeast and in the mutants isolated by M. Grenson, notably to see if this uptake was inhibited by streptomycin. This work was carried out in collaboration with Jacques Béchet, who was an assistant of J.-M. Wiame who was working on arginine anabolism and who also taught microbiology at *Institut Meurice*. A few months later, in May 1962, a first publication authored by J. Béchet, J.-M. Wiame, and M. De Deken-Grenson appeared in the *Archives Internationales de Physiologie et de Biochimie*. At the time it was customary for Belgian researchers to publish in this journal, in abstract form, work that they had presented at scientific meetings organized by the *Société Belge de Biochimie*. The abstract described that in *S. cerevisiae* arginine repressed an enzyme involved in arginine biosynthesis: ornithine transcarbamoylase (OTC), which is the product of the *ARG3* gene. To modulate the intracellular arginine concentration, they grew an arginine auxotroph (strain D20) in continuous culture under arginine limitation, a condition that they found to cause derepressed OTC activity. Arginine addition to a culture of an arginine prototroph (D19) repressed OTC activity. Repression was not observed in the mutant strain D58, believed to be deficient in arginine uptake. A few months later, in October of 1962, J.-M. Wiame, J. Béchet, M. Mousset, and M. De Deken-Grenson published a second abstract in *Archives Internationales de Physiologie et de Biochimie*, entitled “Mise en évidence d’une perméase de l’arginine chez *Saccharomyces cerevisiae*”(translation: “Identification of an arginine permease in *Saccharomyces cerevisiae*”) [17]. This abstract primarily describes the results that were obtained by Madeleine Mousset during her master’s thesis. In this work, the uptake of radioactive arginine was measured in strain D19, a lysine and histidine auxotroph. In strain D58, derived from D19 and selected for its resistance to canavanine, this arginine transport activity was greatly diminished. Strain D58 was found to contain an additional, recessive mutation “segregating in Mendelian fashion in tetrads issued from diploids”. By crossing strain D58 with the arginine auxotroph D20, M. Grenson also observed what seems to have been the first described case of synthetic lethality in yeast: the double mutant that was impaired in both arginine transport and arginine synthesis was unable to grow unless the medium contained about 100 times the amount of arginine normally used to meet the arginine requirement of the auxotroph. The master’s thesis of M. Mousset, presented in October 1962, also describes experiments in which the apparent Km of the permease, its optimal pH, and its specificity were determined. In particular, the simple fact that arginine uptake could be measured in the presence of high concentrations of lysine and histidine (strain D19 being auxotrophic for these amino acids) indicated that the identified permease was specific. This was confirmed by additional experiments that were based on the use of arginine analogs. Furthermore, M. Mousset invalidated the hypothesis from which her research project stemmed, by showing that streptomycin does not inhibit arginine uptake. Lastly, she observed that arginine uptake, measured on cells having been washed and resuspended in arginine-free medium, was reduced when arginine was present in the culture medium. This reduction in transport activity has recently been shown to be at least partially due to the endocytosis of the permease, which was triggered by the transport of its substrate [18,19]. 


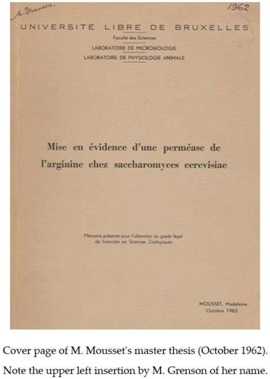


At the time of this initial work on arginine transport in yeast, M. Grenson very often visited the laboratory of J.-M. Wiame, where she participated in discussions on the experiments of M. Mousset and J. Béchet. M. Mousset still remembers those discussions in the office of “the boss”, during which M. Grenson and J.-M. Wiame asserted their very different personalities. M. Grenson, showing quiet reserve, paid special attention to how each experiment was conducted and to the precision of the data obtained. J.-M. Wiame, in contrast, overflowed with ideas for new experiments, with natural and communicative enthusiasm. 


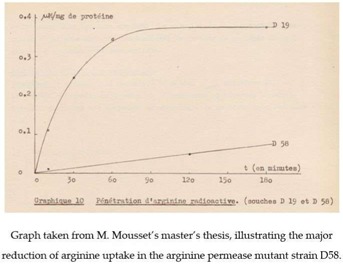


While the focus of M. Grenson and J.-M. Wiame was on nitrogen metabolism in yeast, the work of R. H. De Deken concentrated on the carbon catabolism of this organism, and particularly on the Crabtree effect. As early as 1958, he had contributed to demonstrating by electron microscopy the presence of mitochondria in yeast [20], and as indicated above, he published at the end of 1960 a study on the appearance of “petite” mutations in various yeast species [10]. Shortly afterward, R. H. De Deken developed cancer. He died of this disease in 1966. For M. Grenson, the last years of her husband’s life were very trying. Just before his death, R. H. De Deken published his two famous papers on the Crabtree effect (cited over 500 times) [21,22]. At this time, he was handicapped by his illness, and it is established that M. Grenson greatly assisted him in finalizing these two papers (on which R. H. De Deken appears as sole author). 

## 5. From the “Rouge-Cloître” to the “CERIA” Research Institute

In the early 60s, M. Grenson was appointed Senior Lecturer under Prof. R. Jeener of the ULB. Yet, as her aim was to delve more deeply into the study of amino acid transporters in yeast, she convinced Prof. R. Jeener to let her join the Microbiology Laboratory of J.-M. Wiame. In February of 1963, during a very harsh winter, M. Grenson thus left the *Rouge-Cloître* to join J.-M. Wiame’s team in Anderlecht, on the opposite side of Brussels. Among the few people who followed her in this move were C. Squilbin, who is mentioned above, and Francine Muyldermans, who had been hired in 1961 as a lab assistant in R. Jeener’s group and who later became M. Grenson’s chief technician. 

For M. Grenson and her small team, joining the laboratory of J.-M. Wiame was a great opportunity. On the one hand, there was more space (M. Grenson’s quarters at the *Rouge-Cloître* were limited to two rooms under the roof). On the other hand, the host team was bubbling with activity, thanks to its already impressive size, to the diversity of its microbiology-related research topics, and certainly also to the dynamic nature and the original personality of its leader. Wearing old slippers, J.-M. Wiame was in the daily habit of asking each of his researchers about the work in progress, giving each one the opportunity to appreciate (or not) the smell of the smoke from his pipe (and later, from his *Gauloises bleues* cigarettes). He came to the lab accompanied by his dog Pitou, which members of the team captured one day to give him an anti-flea treatment. J.-M. Wiame’s laboratory at the IRC had rather comfortable financial means and an impressive technical staff for preparing the culture media and sterilizing the glassware that was used each day by the researchers. The technical team also designed, built, and ensured the maintenance of certain laboratory instruments. The laboratory even had a cook, who prepared daily the noon meal for the entire team. This meal was a pleasant get-together, offering many opportunities to discuss ongoing experiments, current events, and other nonscientific topics. M. Grenson was herself a *cordon bleu*, and more than once enchanted the team’s taste buds by sharing recipes of local French cuisine.


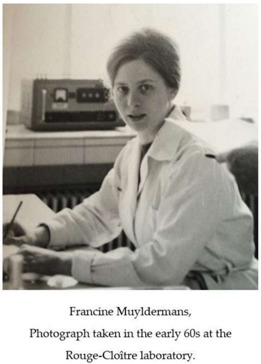


After joining J.-M. Wiame’s team, M. Grenson continued, with her technician F. Muyldermans and the students in her charge, to develop the study of the diversity and regulation of amino acid permeases, a quest that she pursued to the end of her career. In parallel, with her expertise in genetics, she contributed to the various studies conducted by J.-M. Wiame and coworkers. These studies mainly focused on the regulation of the metabolism of nitrogenous compounds (especially arginine) in yeast, including nitrogen catabolite repression. The long-term collaboration J.-M. Wiame and M. Grenson established from the early 60s even extended in their personal lives with their marriage. 

The remainder of this tribute will focus principally on M. Grenson’s work on amino acid permeases. 

## 6. Methodological Improvements

**Figure 1:**
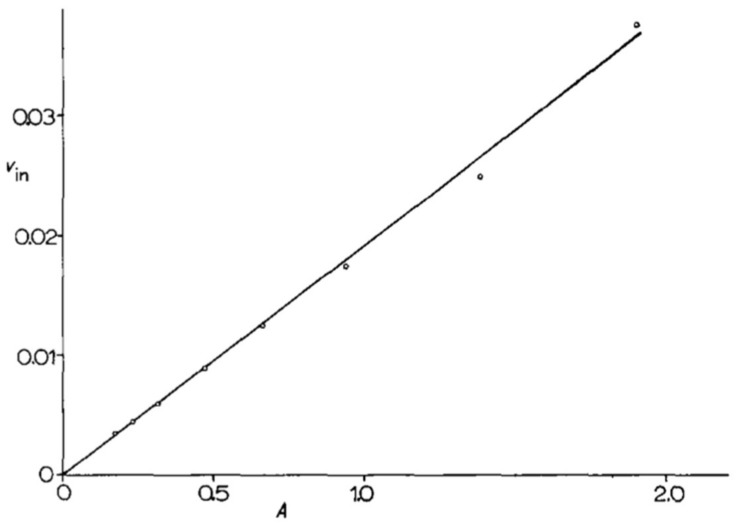
Plot from Grenson et al. (1966) (adapted from reference [24] with permission from Elsevier) illustrating that the initial [^14^C]-arginine (0.2 mM) uptake rate (V_in_, expressed in micromoles of arginine incorporated per minute and per milliliter) measured during balanced growth increases proportionally to the optical density of the culture (*A*).

At the very beginning of their collaboration, M. Grenson and J-M. Wiame defined a set of methods to be applied in all future work. They paid special attention to yeast cultures, defining citrate-buffered minimal media (citrate cannot be used as a carbon source by yeast cells) with a precise composition, and choosing to perform experimental measurements on cells that were harvested in the exponential phase, a significant number of generations after seeding. Taking these precautions was found to make their experimental measurements highly reproducible. In one of her first publications, for instance, M. Grenson measured the uptake of a ^14^C-labeled amino acid in six independent experiments (biological replicates) and found the uptake rate to vary by only a few percent. In another experiment that was inspired by the work of J. Monod [23], she measured the activity of the arginine permease at different time points during the exponential growth phase and plotted the measured activities, expressed per ml of culture, versus the optical density of the culture (differential rate of synthesis). The activity values were found to fall almost perfectly on a straight line that could be extrapolated to the origin. This is proof of the great stability of cells during the balanced phase of exponential growth (Figure 1).

J.-M. Wiame and M. Grenson also found it indispensable to define a new wild-type reference strain having no auxotrophic mutations, to be used to derive all of the mutant strains. There was a problem with the mutant strain D58, isolated in 1962 from a histidine and lysine auxotroph (strain D19), as obtained by mutagenesis of strain YFa (YF stands for “Yeast Foam”): M. Grenson had quickly realized that the amino acids added to the medium to meet the nutritional requirements of this auxotrophic strain could sometimes interfere with measurements of labeled amino acid uptake. Also, the YF diploid appeared to be heterogenic (after sporulation, the strain did not always yield the four expected spores). A new, prototrophic reference strain was thus isolated: strain Σ1278b. It was obtained by first crossing the YFa-derived yeast D77 (auxotrophic for uracil and glutamate) with the yeast 1422-11D that was received from the American geneticist Donald C. Hawthorne. The derived haploid strain Σ15d (Σ stands for “segregant”) was then crossed with strain DP1-1B received from Piotr Slonimski, and one of the spores issued from this cross gave rise to Σ1278b. This strain is well known to the yeast research community. One reason for this is that, unlike most wild-type *S. cerevisiae* strains, Σ1278b bears no mutations impairing the natural phenomenon of pseudohyphal growth displayed by diploid yeast strains on nutritionally poor medium. This particular type of growth was rediscovered in the early nineties by Carlos Gimeno and Per Ljungdahl of the laboratory of Gerry Fink [25], when they analyzed an *apf* strain that M. Grenson had sent to G. Fink twenty years earlier (see note in Appendix A). Strain Σ1278b has yet further advantages. For example, the group of P. Agre showed in 2001 that it is one of the rare laboratory strains whose aquaporin genes are functional because they contain no mutations [26]. Another, much earlier observation (made by M. Grenson) was that strain Σ1278b displays an inhibition of a particular permease activity when it was grown in the presence of ammonium, which is a good nitrogen source (the permease later turned out to be the general amino acid permease Gap1). This regulation is absent or at least much weaker in other strains, such as strain YFa, which was used in M. Grenson’s early work. The yeast strains used to this day in some laboratories are known to contain one or several recessive mutations impairing this regulation by ammonium, known today to involve the Target Of Rapamycin kinase Complex I (TORC1) [27]. I have found in the archives of M. Grenson a letter she sent in 1989 to her friends Piotr Slonimski and André Goffeau. In the letter, convinced of the advantages of strain Σ1278b and enclosing supportive documents, she tried to convince them to use Σ1278b as reference strain in the yeast genome sequencing project. Her suggestion was not followed, but the genome of strain Σ1278b was later sequenced by the group of Charlie Boone [28]. 

## 7. Early Work on Amino Acid Transport: Identification of the Specific Arginine (Can1), Lysine (Lyp1), and Methionine (Mup1) Permeases

Although the first permease mutant that was impaired in arginine uptake (strain D58) was isolated in 1962, M. Grenson’s paper describing a specific arginine permease in yeast was not published until four years later [24]. This delay may be due to multiple factors: the time it took M. Grenson to get settled in J.-M. Wiame’s group and the time that she devoted to the above-mentioned methodological improvements, to finalizing the two papers of R. H. De Deken, to teaching (her appointment at the ULB led to new teaching duties), and to supervising her students. These included C. Squilbin and M. Mousset, the latter having begun a doctorate in 1962. In early 1965, biologists Jacqueline Gits and Claude Joiris joined the team in the framework of their master’s theses. J. Gits studied valine transport and C. Joiris studied glutamate, aspartate, and α-aminoadipate transport. Both began doctoral research in October 1965. M. Grenson also devoted considerable time to training Francine Muyldermans. When hired as a lab assistant in December 1961, F. Muyldermans had been working in a chocolate factory and had no technical or scientific qualifications. M. Grenson is the person who taught her basic chemistry and the various techniques of microbiology and yeast genetics. Thanks to this training, and also to her sound common sense and hard-working organized nature, F. Muyldermans made major contributions to all the scientific work of M. Grenson and students over a period of 25 years. She remained M. Grenson’s technician until her early retirement in 1989. It was F. Muyldermans who dissected ascus no. 1278, whose second spore (b) yielded the lab’s new reference strain Σ1278b. Shortly afterward, she isolated the arginine permease mutant, MG168, by culturing the mutagenized Σ1278b on medium containing the toxic arginine analog canavanine. Applying the same procedure, but using the lysine analog thiosine instead of canavanine, she isolated the first lysine permease mutants. 


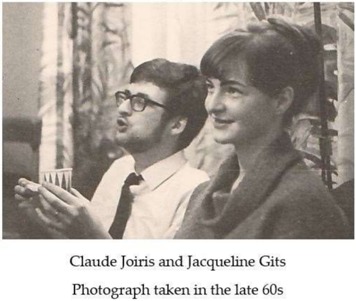


**Figure 2:**
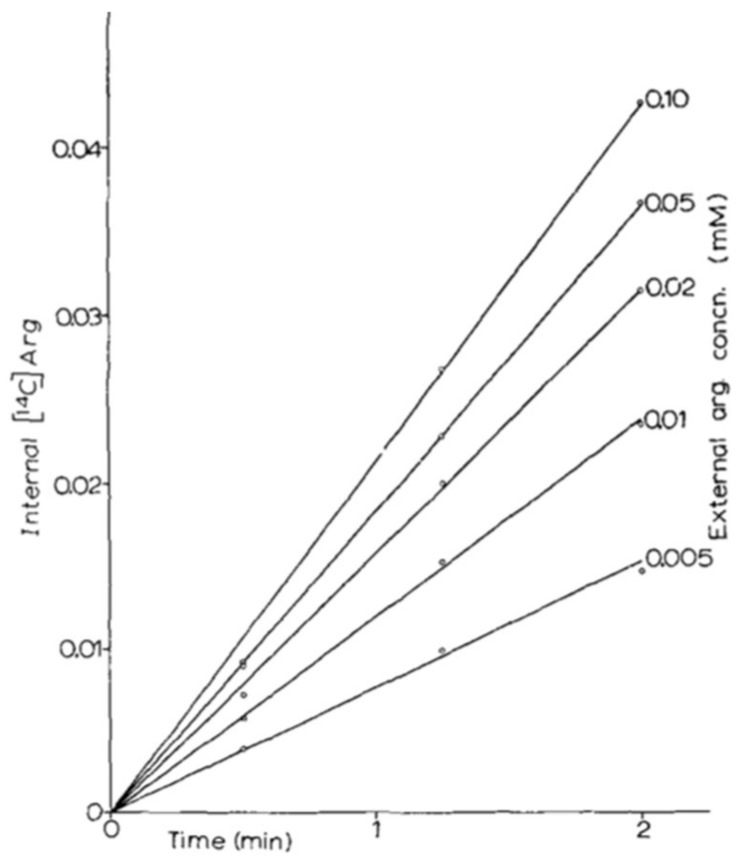
Plot from Grenson et al. (1966) (adapted from reference [24] with permission from Elsevier), illustrating the uptake of [^14^C]-arginine over time when the external arginine concentration was varied.

It was thus in 1966 that two papers were published side by side in *Biochim. Biophys. Acta*: one on the arginine permease [24] and one on the lysine permease [29]. These two pioneering papers illustrate well the methodology that was developed by M. Grenson, applied in all of her work on yeast permeases and still used to this day in my laboratory. The prototrophic strain Σ1278b was grown on citrate-buffered minimal medium containing ammonium as sole nitrogen source, and thus devoid of amino acids. To measure the permease activity, labeled substrate was added directly to a sample of culture in balanced growth. At different times, 1-mL aliquots were taken. The cells were recovered by filtration and a quick wash on nitrocellulose prior to measuring the incorporated radioactivity. By varying the external concentration of substrate (Figure 2), it was possible to measure the apparent *K*_m_ of the permease and its uptake rate at saturation (*V*_max_), which is expressed in nanomoles of substrate incorporated per minute and per milligram of protein. 

In a Lineweaver-Burk plot, it is also possible to show that several permeases are involved in the uptake of an amino acid, provided that they have sufficiently different *K*_m_ and *V*_max_ values. This occurs, for example, with lysine uptake, the permease with the lower affinity having turned out to be the arginine permease. 

The specificity of each permease is tested by measuring the uptake of the radioactive amino acid in the presence of another amino acid or of an analog of the substrate amino acid. When inhibition occurs, the experiment is repeated with different concentrations of transported substrate and unlabeled inhibitor to see if the inhibition is competitive. If so, the measured inhibition constant Ki provides an indirect measurement of the apparent affinity of the permease for the inhibitor. This is particularly useful when the inhibitor is not available in labeled form. To determine whether it is the radioactive substrate itself or a derivative thereof that accumulates in cells (see note in Appendix B), the metabolites are extracted from the cells and the electrophoretic mobilities of the extracted radioactive compounds are compared with that of the pure labeled amino acid. The extracted metabolites can also be added to cells that are doubly auxotrophic for the tested amino acid (to avoid reversion of auxotrophic phenotype), so as to measure the resulting biomass gain. By comparing this gain with those that were observed upon adding known quantities of the amino acid, it is possible to precisely measure the amount of amino acid absorbed (bioassay). 

A few months after the joint publication of the papers describing the arginine and lysine permeases, a third study that was based on the same methodology was published, in which J. Gits and M. Grenson demonstrate the existence of two methionine-specific permeases, a high-affinity and a low-affinity permease [30]. The paper notably describes mutants that are impaired in the high-affinity permease, selected for their resistance to the toxic methionine analog l-ethionine (see note in Appendix C). 

Today, the permeases that are described in these three papers are well known. They are encoded by the genes *CAN1* (arginine permease) [31], *LYP1* (lysine permease) [32], *MUP1,* and *MUP3* (respectively, the high- and the low-affinity methionine permease) [33]. To name yeast genes, M. Grenson and J.-M. Wiame adopted the nomenclature used by Milislav Demerec for bacterial genes. The permeases just mentioned thus received the names arg-p1, lys-p1, met-p1, and met-p2, respectively. This choice of nomenclature, which M. Grenson and J.-M. Wiame later abandoned, turned out to be somewhat unlucky, as some of their work is not readily identified on the basis of the nomenclature used today. 

## 8. Exploring the Diversity of Amino Acid Permeases and First Observations on Their Regulation

The ten years following the integration of M. Grenson into the IRC were one of the most fruitful periods in her scientific career, which are summarized hereunder. This period coincided with the arrival of several new students. In early 1967, the chemistry students Claire Hou and Marjolaine Crabeel devoted their master’s theses to studying the inhibition of amino acid uptake by ammonium. Both of them went on to do a doctorate. The doctoral thesis of C. Hou focused on characterizing the general amino acid permease (Gap1) and its inhibition by ammonium, and that of M. Crabeel dealt with the inhibition of permeases by their own substrates (feedback inhibition). C. Hou and M. Crabeel made major contributions to the first publications of M. Grenson’s group on permease downregulation. In early 1968, zoology student Claudette Hennaut also joined the lab, closely followed by Claude Darte and Christian Casteleyn. All three also undertook doctoral studies. By the late 60s or early 70s the team, with its almost yearly addition of one or several master’s students, reached a quite impressive size that favoured rich scientific exchanges. The early 70s, however, were also marked by less fortunate events. In September of 1970, M. Grenson was injured in a serious automobile accident, as a passenger, during a trip with colleagues. She incurred serious damage to her leg and her convalescence lasted several months. “She will never walk again”, said J.-M. Wiame shortly after the accident. For a long time after her return to the laboratory, M. Grenson walked with a cane. By the mid-70s, her group counted considerably fewer students. This is the time when, in the spring of 1975, M. Grenson decided to end her personal relationship with J.-M. Wiame. Within the laboratory, she then moved from the space that she had shared for twelve years with J.-M. Wiame to a more remote location of the IRC. This separation, affecting both her personal and professional lives, did not prevent M. Grenson and J.-M. Wiame from continuing to collaborate scientifically. A few years later, in 1983, M. Grenson left the IRC permanently to create a new laboratory on the ULB Campus of the Plain in Ixelles. As will be described later, the establishment of her new laboratory in Ixelles marked the beginning of a new period in her scientific career.


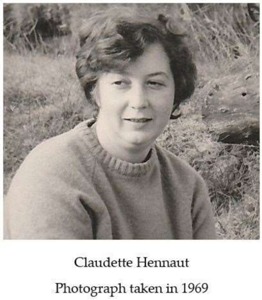


The next section of this tribute deals with the studies carried out by M. Grenson’s team at the IRC (prior to her move to Ixelles), where the work was primarily focused on permease diversity and regulation. A subsequent section describes the research that was conducted after her move to the campus in Ixelles, marked by the first applications of gene cloning technologies in her laboratory and by an ambitious collaboration with an industrial group. 

### 8.1. Identification of a General Factor (Apf1/Shr3) Required for the Activity of Various Amino Acid Permeases

At the 55th meeting of the *Société belge de Biochimie* held in Leuven on 22 October, 1966, M. Grenson was invited to present her ongoing work on “The transport of metabolites—particularly amino acids—through cell membranes”. Detailed proceedings of this conference were published in the *Revue des Fermentations et des Industries Alimentaires*. In them, M. Grenson summarizes the views of the time on amino acid transport. According to the model retained, the transmembrane transport of an amino acid would take place in at least two steps, which was catalyzed by different proteins: the first protein, the permease, would bind the substrate present in the external medium with a certain selectivity and affinity. Then the substrate would be passed on to a less specific “transport protein” that would ensure translocation of the amino acid. It might be useful to stress that this model was imagined at a time when the membrane was still viewed as two layers of proteins that were separated by a lipid bilayer.

Today, it may seem strange to imagine that in addition to the permease, a second protein with lesser specificity might intervene in the uptake of various amino acids, although ATP-binding cassette (ABC) importers from gram-negative bacteria utilize a substrate-binding protein (SBP) to deliver substrates to the cognate transporter [34]. The assumption that a permease cooperates with a poorly selective translocator was inspired from results, obtained very early by M. Grenson’s team and other laboratories, showing that it is possible to isolate yeast mutants impaired in the uptake of many amino acids. This type of observation is what led the groups of De Robichon-Szulmajster (Gif-sur-Yvette, France) and L. W. Parks (Oregon, USA) to propose in 1964–1965 that yeast possessed only one permease for all amino acids [35,36]. Yet the first results of M. Grenson’s group, obtained already in 1962, showed on the contrary that each permease appeared highly specific. This suggested that mutations affecting the uptake of several amino acids (called *apf*, for *amino acid permeability factor*, in the group of M. Grenson) must alter more general factors required for the transport activity of several permeases, for example, a factor that is required to translocate an amino acid that is recognized beforehand by a permease. At the end of the discussion section of her 1966 publication on the arginine permease, M. Grenson thus mentions isolation of a mutant (strain RA68) having the same properties as those that are described by De Robichon-Szulmajster and Parks. In 1971, M. Grenson and C. Hennaut published a paper describing more completely this mutant, called *apf1*, which was isolated like other *apf* strains for its resistance to several toxic amino acid analogs and its inability to use each of various amino acids as a sole nitrogen source [37]. This study showed that this mutation specifically affects the Vmax values of amino acid permeases (their respective apparent Km values remain unchanged), without altering the transport of other nitrogenous compounds (uracil, uridine, cytosine). The mutation, allelic with the *aap* mutation that was isolated in 1965 by Yolande Surdin-Kerjan in the laboratory of De Robichon-Szulmajster, affects a gene that was described twenty years later, called *SHR3* by Per Ljungdahl and collaborators [38]. Today, thanks to the work conducted in Stockholm by P. Ljungdahl’s team, we know that this gene codes for an endoplasmic reticulum (ER) protein that functions early in the secretory pathway to facilitate the co-translational folding of amino acid permeases. Apf1/Shr3 belongs to a quite diversified class of ER membrane-localized chaperones that facilitate the correct folding of discrete sets of polytopic membrane proteins, which is a requisite for their inclusion into COPII-coated transport vesicles [39]. 

### 8.2. Identification of the General Amino Acid Permease (Gap1) 

While characterizing the specific arginine, lysine, and methionine permeases, M. Grenson and students had noticed that when a mutant impaired in one of these permeases was transferred to conditions of nitrogen starvation, there reappeared a transport activity for the relevant substrate. This activity was also present when the mutant was grown on a medium containing proline, which is a poor nitrogen source, instead of ammonium, the optimal nitrogen source that is usually present in the growth medium. This unexpected fact is already recorded in the master’s thesis of M. Mousset (October 1962), who found that an *arg-p1* (*can1*) mutant becomes sensitive to canavanine if it is grown on a medium containing a poorer nitrogen source such as glutamate, and that the synthetic lethality caused by combining the *arg-p1* (*can1*) mutation with a mutation causing arginine auxotrophy is no longer observed when ammonium is replaced with glutamate. The same type of result was later obtained in the case of the lysine and methionine uptake activities. In her conference presentation in 1966 (see above), M. Grenson already concluded that “what we are witnessing here is a regulatory function affecting amino acid uptake in yeast, especially since ammonium is the best nitrogen source for the yeasts we use”. M. Grenson’s team also observed that this inhibition of amino acid uptake by ammonium is absent or less pronounced in strains other than Σ1278b, such as strain YFa used in the first experiments. Shortly afterward, C. Hou began work towards her doctorate. During this work she showed, with the help of M. Crabeel, that the various transport activities appearing in the absence of ammonium are due to a single protein, a general amino acid permease, which is thus inhibited in the presence of ammonium. The best way to detect the activity of this permease is to grow the cells on a medium containing proline as nitrogen source and to measure the uptake of radiolabeled citrulline or tryptophan, which is negligible in ammonia-grown cells. The uptake of each of these amino acids is competitively inhibited by the other, and the same applies to other amino acids. To isolate mutants impaired in this permease, F. Muyldermans isolated from the arginine-permease-deficient strain MG168 a clone having become resistant to canavanine on proline medium. After crossing, this clone yielded strain 2512c, carrying a single mutation: a *gap* mutation. In this strain, no citrulline or tryptophan transport activity appears when ammonium is replaced with proline as a nitrogen source. In agreement with the idea that the *gap* mutation alters the structure of the general amino acid permease, M. Crabeel showed that a mutation located at the same genetic locus affects the affinity of the permease for its substrates. The paper describing the existence of this general amino acid permease in yeast was published in 1970 in the *Journal of Bacteriology* [40]. To date, it has been cited more than 300 times. The Gap1 permease is one of the yeast plasma-membrane proteins that has been most intensely studied to this day by many laboratories. The mechanism that is underlying the inhibition of this permease by ammonium was to become one of the main research topics of M. Grenson’s group (see below). 


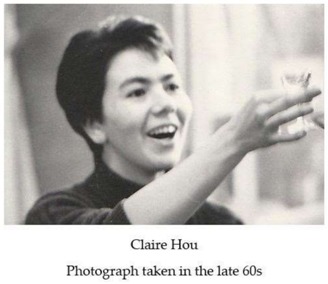


### 8.3. Identification of Other Amino Acid Permeases

Several other amino acid uptake activities were studied by students of M. Grenson, from the mid-60s onward. For example, Carla Dubbeling, a master’s student who was supervised by M. Crabeel, measured two histidine uptake activities, a high- and a low-affinity activity, during her master’s thesis in 1967. A first attempt to isolate mutants that were deficient in these permeases was made by selecting methylhistidine-resistant mutants, but the mutants obtained showed no impairment of histidine transport. The same was true of mutants that were able to grow in the presence of toxic concentrations of histidine. M. Crabeel, who pursued during her doctorate the study of histidine transport, applied a third method: selecting, from a histidine-auxotrophic strain, mutants that were unable to grow in the presence of histidine supplied at low concentration to compensate for the auxotrophy. She thus obtained a *his-p1* mutant that was impaired in the high-affinity permease. In 1970, M. Crabeel and M. Grenson published a paper describing the existence of a specific histidine permease [41], later named Hip1. Shortly afterward, M. Grenson and J.-M. Wiame visited Gerry Fink, then a young assistant professor at Cornell, who was studying histidine metabolism in yeast. As a result of this encounter, M. Grenson sent G. Fink the mutant *his-p1* along with other strains. Fifteen years later, when genetic engineering techniques became accessible, this mutant was exploited by G. Fink’s group to clone by complementation, and then sequence the *HIP1* gene, encoding the specific histidine permease [42]. This study was based on the methodology that was developed already in 1979 by J. Broach, J. Strathern, and J. B. Hicks (Cold Spring Harbor) to isolate *CAN1*, which is one of the first cloned yeast genes [43]. The genes *HIP1* and *CAN1*, sequenced almost simultaneously, were the very first genes encoding plasma membrane transporters to be characterized at the molecular level in yeast. 


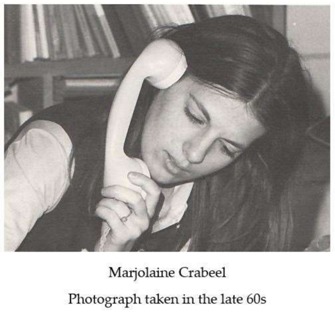


Claude Joiris, who joined M. Grenson’s group in 1965, studied glutamate and aspartate transport, and this work was continued by Claude Darte from 1972 onward. It had been observed that a lysine auxotrophy could be compensated for by α-aminoadipic acid, which is a precursor of lysine in its biosynthesis pathway. Having found the uptake of α-aminoadipic acid to be competitively inhibited by glutamate, C. Joiris went on to show that this compound enters cells via a high-affinity dicarboxylic acid permease. Mutants that were impaired in this permease were obtained by isolating, from a lysine auxotroph, mutants unable to use α-aminoadipic acid as an external source of lysine. When this mutant is grown on ammonium, it is unable to take up glutamate, aspartate, or α-aminoadipate. The *dic-p1* mutation present in this strain affects the gene, which was later to be named *DIP5*, encoding a dicarboxylic acid permease [44]. 

The study of valine uptake was undertaken in early 1965 by J. Gits, whose doctorate focused on the transport of the methylated amino acids (methionine, leucine, valine, isoleucine, alanine, and threonine). This study turned out to be much more complex than those that were performed in the laboratory on other amino acids. In fact, today we know that the transport of each of these amino acids involves several permeases (Bap2, Bap3, Agp1, Gnp1…), showing fairly low specificity and low affinity. Furthermore, the synthesis of these permeases is induced, to different extents, in response to the presence of various substrate amino acids. This set of properties was evidenced in the thesis of J. Gits, but the results, published in an abstract [45], were never published in an international journal, perhaps because M. Grenson felt that the complexity of the transport of this group of amino acids warranted an in-depth study. Today, we know that the genes encoding these permeases are transcriptionally induced upon detection of external amino acids by the plasma-membrane sensor Ssy1, which is a protein homologous to amino acid transporters, but is lacking transport activity [46,47,48]. Among the various *apf* mutants that were isolated by M. Grenson’s group, and particularly by C. Casteleyn, one (*apf3*) later turned out to be affected in the *PTR3* gene [49], encoding the protein which acts just downstream from Ssy1 in the transduction pathway activated upon detection of external amino acids [50,51].

M. Grenson’s group studied the transport of yet other amino acids, but no publications arose from this work. Examples include the study aromatic amino acid transport by C. Hennaut (1968) and of proline transport by Gabriel Solbu (1974), a master’s student. 

### 8.4. Study of Uracil, Cytosine, and Uridine Uptake

François Lacroute, when working in the early 60s as a graduate student under P. Slonimski, visited J.-M. Wiame’s laboratory several times. He notably studied with M. Grenson and André Piérard the biosynthesis of carbamoyl phosphate, which is biosynthetic precursor of arginine and pyrimidines [52]. His thesis work also led him to isolate mutants resistant to 5-fluorouracil (see note in Appendix D) and to show that some of them contained a mutation affecting uracil transport. He concluded that this mutation affected a gene encoding a pyrimidine permease. This study was continued by M. Grenson, who published in 1969 a paper on the transport of uracil, cytosine, and uridine [53]. She showed that each of these compounds is taken up by a specific permease that can be inactivated selectively by a *ura-p* (for uracil), *cyt-p* (for cytosine), or *urid-p* mutation. The *cyt-p* mutants were isolated for their inability to use cytosine as sole nitrogen source, and the *ura-p* and *uri-p* mutants for their resistance to 5-fluorouracil and 5-fluorouridine, respectively. These mutations were also isolated shortly afterward by F. Lacroute. They alter the genes that he named *FUR4* (uracil permease), *FCY2* (cytosine permease), and *FUI1* (uridine permease) [54]. As described below, this work of M. Grenson also revealed a negative regulatory mechanism called feedback, affecting the uracil and uridine permeases and that was observable when the substrates of these permeases accumulate inside the cell. In a later study published in 1973, Annemarie Polak and M. Grenson reported that the transport of adenine and hypoxanthine is also mediated by the Fcy2 permease [55]. 

### 8.5. Study of Ammonium Transport

Ammonium is a preferential nitrogen source for yeast, particularly for strain Σ1278b. In its presence, the Gap1 permease is inhibited, and in some mutants isolated by M. Grenson’s group, this negative regulation is relieved (see below). Inhibition of ammonium transport was envisaged as a possible cause of this relief. These observations and others prompted M. Grenson and her team to identify permeases for the nitrogenous ion. Earlier work by R. J. Roon et al. had shown that methylamine is a toxic ammonium analog whose uptake into yeast is competitively inhibited by ammonium [56]. This suggested that at least one permease might intervene in the transport of both of the compounds. C. Hennaut therefore isolated methylamine-resistant mutants. Among these, strain CH915 showed an interesting phenotype: its growth rate was considerably reduced on a medium containing ammonium at low concentration as sole nitrogen source. Later, genetic analysis of this strain revealed the presence of two mutations, named *mep-1* and *mep-2*, each causing the loss of one methylamine uptake activity, a low-affinity activity (*mep-1*), and a high-affinity activity (*mep-2*). As both of the activities were competitively inhibited by ammonium, the subsequent measurement of the Ki values provided an indirect measurement of the affinity of each permease for ammonium. This pioneering work on ammonium transport was published in 1979 [57], with Evelyne Dubois as the first author. After completing her chemistry studies at ULB, E. Dubois had carried out her doctoral studies in J.-M. Wiame’s laboratory. She collaborated closely with M. Grenson for a long time, particularly on ammonium transport and nitrogen catabolite repression. 


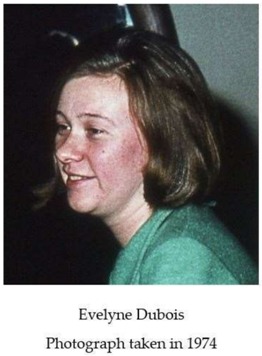


Fifteen years later, the work of A.M. Marini, which was carried out during her doctorate, revealed that the mutations that were present in the strain isolated by C. Hennaut and studied by E. Dubois affected the genes *MEP1* and *MEP2*, coding for plasma-membrane ammonium transporters that are conserved in all kingdoms of life [58,59]. Unexpectedly, furthermore, these proteins turned out to be homologous to the human Rhesus (Rh) blood-group factors, whose role in red blood cells was not known [60]. It turned out that the Rh proteins, some of which are expressed in the kidneys, also function as ammonium transport proteins, an activity shown initially by expressing them in yeast [61]. There exists in yeast a third ammonium permease gene, *MEP3*, which was capable of supporting growth on a low-ammonium medium. The reason why the *mep1 mep2* double mutant described in 1979 showed a growth defect despite the presence of Mep3 was elucidated when it was shown that the mutant protein that was encoded by the *mep-1* allele has a dominant-negative effect on the activity of Mep3 [62]. The explanation of this phenomenon, as reported by the group of Wolf Frommer, is that the ammonium transporters associate into complexes within which each subunit exerts, via its cytosolic C-terminal region, an allosteric regulation on the activity of the neighboring subunit [63]. 

### 8.6. Permeases as Internal-Metabolite-Retaining Systems

An arginine auxotroph placed in arginine-free solid medium can grow if it is close to a *gap1 can1* mutant impaired in arginine uptake, but not if it is close to a wild-type strain. This shows that a *gap1 can1* mutant excretes arginine. This cross-feeding was reported for the first time in a paper by M. Grenson, published in 1973 [64]. M. Grenson observed this excretion phenomenon with other amino acids and deduced that an important role of permeases, especially high-affinity ones, is to ensure that amino acids are retained in the cell. This is an important notion that is often neglected when the physiological role of permeases is mentioned. To analyze this excretion phenomenon, M. Grenson isolated mutant strains excreting abnormally large quantities of one or several amino acids. Among these mutants she found mainly auxotrophic or bradytrophic strains. She also observed that cells that take up certain intermediates in the biosynthesis of particular amino acids excrete those amino acids into the medium. In another paper, she reports that uracil and cytosine excretion by certain mutants affected in pyrimidine metabolism [53], thus extending observations that were previously reported by her colleagues F. Lacroute and P. Slonimski. The experiments on amino acid excretion were to constitute the basis of the collaboration that M. Grenson and coworkers established with an industrial consortium shortly before moving to the laboratory that was located on the campus in Ixelles (see below). They also inspired a study that we published in 2004, on the identification and characterization of a yeast protein that was capable of excreting various amino acids [65]. According to the model that is described in the published report, this transporter (Aqr1) would be located at the level of small internal vesicles, thus enabling them to accumulate amino acids that are present in excess in the cytoplasm. These vesicles, upon fusing with the plasma membrane, would release the excess amino acids into the external medium, just as neurons release neurotransmitters (some of which are amino acids) by exocytosis. The Aqr1 protein, furthermore, shares sequence similarities with certain neurotransmitter transporters that are present at synaptic membranes. It is thus tempting to imagine that a system ensuring, in certain organisms, the excretion by exocytosis of metabolites that are present in excess in the cytosol, became in the course of evolution a specialized intercellular communication system on which the synaptic transmission between nerve cells is now based [65]. 

## 9. Regulation of Amino Acid Permeases

From the start of their work on amino acid permeases, M. Grenson and students accumulated data indicating that these proteins are regulated according to the composition of the medium as regards nitrogenous compounds. They soon realized that the regulatory mechanisms, which can be inhibitory or stimulatory, can affect either permease synthesis, permease activity, or both. To distinguish these two effects, M. Grenson’s group made much use of the graphic representation method that was described by J. Monod, where the activity of an enzyme or permease, expressed per unit volume of culture, is monitored during growth [23], like in the experiment that is shown in Figure 1. When the cells are shifted from one condition to another, it is possible to see if the observed activity change is due to regulation of the protein’s synthesis or activity.

The study of permease regulation rapidly became one of M. Grenson’s main research topics. These analyses contributed to revealing the mechanisms, well known today, that intervene in the regulation of membrane transporters in all organisms and whose dysfunctioning is sometimes associated with pathologies. 

### 9.1. Inhibition of the Gap1 Permease by Ammonium Ions

**Figure 3:**
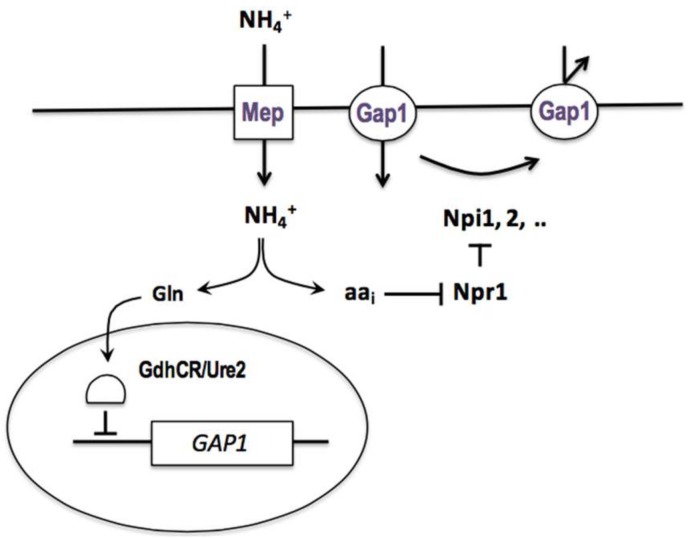
Model of Gap1 permease downregulation in the presence of ammonium, proposed in 1983 by M. Grenson. The regulation affects expression of the *GAP1* gene and hence the synthesis of the Gap1 permease, and it also affects the activity of the protein present in the plasma membrane [66].

While working towards her master’s degree in 1962, M. Mousset observed that an arginine transport activity reappeared in the *can1* mutant upon its transfer from an ammonium-containing medium to a nitrogen-poor medium. The same phenomenon was observed shortly afterward with mutants that were defective in the lysine or methionine permease. In competition experiments, C. Hou showed during her doctorate that these different uptake activities appearing on nitrogen-poor media are all due to the same permease, the general amino acid permease (*gap*), which is inhibited by ammonium (see above). She undertook to study this inhibition and found it to be noncompetitive, to require the penetration of ammonium into the cells, and to principally affect the activity of the permease. To study this novel regulatory phenomenon, M. Grenson looked for mutants where the Gap1 permease was active despite the presence of ammonium. This analysis was complicated by the fact that two mechanisms contribute to the loss of Gap1 activity in the presence of ammonium: repression of the synthesis of the permease and inhibition of its activity, and that both mechanisms must be neutralized to render Gap1 fully active in the presence of ammonium. After about fifteen years of research that I broadly outline in an endnote (Appendix E), M. Grenson and coworkers succeeded in clearly revealing this dual downregulation of Gap1 and in identifying genes involved in each mechanism. In an outstanding paper published in 1983 [66], M. Grenson integrated all of these observations to propose a general model of Gap1 regulation by ammonium (Figure 3). According to this model, the *GAP1* gene is repressed in the presence of ammonium, and this repression is relieved in cells that are mutated in the *URE2* gene (initially named *GDHCR* by E. Dubois and M. Grenson) and in cells where glutamine synthesis is deficient. In wild-type cells that are grown on a poor nitrogen source, such as proline or urea, this repression is not exerted, and the permease is synthesized and highly active. If ammonium is added to these cells, *GAP1* expression is repressed, and the presynthesized Gap1 molecules are rapidly inactivated. This inactivation requires the products of several genes, called *NPI* (Nitrogen Permease Inactivator), whose action is stimulated by the conversion of ammonium to amino acids. Some mutant forms of Gap1, such as Gap1(pgr), are insensitive to Npi-mediated inhibition of Gap1 activity. Furthermore, this inhibition can potentially function even in cells growing on a poor nitrogen source, but it is effectively prevented by a protein called Npr1 (Nitrogen Permease Reactivator), which thus acts as a positive regulator of Gap1 activity. Upon the addition of ammonium, this protective effect of Npr1 is suppressed, and the Npi factors are therefore able to inhibit the permease (Figure 3). What is more, this complex regulation involving the Npr1 and Npi proteins controls the activity of other permeases, such as the proline permease (Put4) and the ureidosuccinic acid permease (Dal5) [66]. 

This regulatory system was later studied at the molecular level, and we know today that it involves mechanisms that are conserved all the way to human cells. The loss of Gap1 activity upon ammonium addition actually results from its selective removal from the plasma membrane by sorting into endocytotic vesicles [67]. Npi1 is the ubiquitin ligase Rsp5, which catalyzes the linkage of ubiquitin molecules to a cytosolic part of the Gap1 permease [67,68,69]. These ubiquitins provide a signal that is triggering both the endocytosis of the protein and its progression through the various compartments of the endocytotic pathway to the vacuole, where it is degraded. Whereas, Npi1/Rps5 triggers Gap1 endocytosis, the other identified Npi factors, Npi2/Doa4 and Npi3/Bro1, intervene at later steps of the endocytotic pathway; in their absence, the permease is normally internalized into vesicles, but it does not progress normally along the endocytotic pathway and it tends to cycle back to the plasma membrane [70,71]. The Npr1 factor is a protein kinase (see below) that does not directly target the Npi factors, but rather two accessory proteins, Bul1 and Bul2, which is required by the ubiquitin ligase Npi1/Rsp5 [27,69]. As these two proteins are functionally redundant, M. Grenson’s group failed to identify them by genetic screening. They belong to the alpha-arrestin family and act as adaptors enabling Npi1/Rsp5 to bind to Gap1. On nitrogen-poor media, the Npr1 kinase is active and phosphorylates the Bul adaptors, thus inactivating them (because like many phosphorylated proteins, the phosphorylated Bul proteins bind to the 14-3-3 proteins). Therefore, Npi1/Rsp5 is unable to catalyze Gap1 ubiquitylation. On the other hand, when ammonium is supplied to the cells and is converted to amino acids, the Npr1 kinase is inactivated, and the Bul proteins become able to recruit the Npi1/Rsp5 ubiquitin ligase to Gap1. The permease is thus ubiquitylated and targeted to the endocytotic pathway [27]. Current studies suggest that the endocytotic process involving the Npi1/Rsp5 ubiquitin ligase and alpha-arrestin-type adaptors may act on all of the proteins that are present in the yeast plasma membrane, and that homologous mechanisms exist in more complex eukaryotic cells [72,73]. Regarding the inactivation of the Npr1 kinase by internal amino acids, the group of Michael Hall has shown that it is promoted by phosphorylation via TORC1, the TOR (Target of Rapamycin) kinase complex 1 [74]. 

### 9.2. Regulation of the Intrinsic Activity of the Ammonium Permeases 

Once the *npr1* mutants were isolated, they were found to display, in addition to a loss of Gap1 and other amino acid permease activities, drastically reduced ammonium uptake [75]. E. Dubois and M. Grenson then found that when cells are grown on a good nitrogen source (e.g., glutamine), the ammonium permeases are subject, like Gap1, to dual negative regulatory mechanisms that are affecting their synthesis and activity. Yet surprisingly, although the activity of the ammonium permeases turned out to be regulated by Npr1, they appeared not to be regulated by the Npi factors. This suggested that their negative regulation involves mechanisms differing from that controlling Gap1. Later, a mutation in a new gene, named *AMU1*, was isolated based on its ability to restore rapid growth of an *npr1* mutant on ammonium medium [57]. 

Much later, as a doctoral student working on ammonium permeases, A.-M. Marini cloned the gene *AMU1* and showed that it encodes a peculiar protein containing a repeated motif. Recently, A.-M. Marini’s group showed that Amu1/Par32 functions as an inhibitor of the Mep1 and Mep3 permeases, and that Amu1 is itself inhibited by phosphorylation by the Npr1 kinase [76]. The mechanism of Mep2 regulation is different, as it does not involve the Amu1 protein. In this case, the permease seems to be directly phosphorylated by Npr1, and thereby activated [77]. In conclusion, the observed reduction in ammonium uptake via the Mep permeases is due to inhibition of the intrinsic activity of these permeases and not to endocytosis, as in the case of Gap1 [76]. 

### 9.3. Feedback Inhibition of Permeases by Their Own Substrates and the Effect of Cycloheximide

Once it was established that Gap1 is inhibited by ammonium, the hypothesis was envisaged that the permease might be subject to feedback inhibition by amino acids accumulating as a result of ammonium uptake, i.e. by internal substrates of the permease. This type of negative regulation might likewise explain why the intracellular accumulation of an externally supplied radiolabeled amino acid remains linear for only a short time: past this time, the amino acid would begin to exert feedback inhibition on the permease. During her doctorate, M. Crabeel studied this feedback inhibition, while using the specific histidine permease Hip1 and the Gap1 permease as reference proteins. She showed that inhibition of each of these two permeases is observed only in the presence of its substrates, that it is reversible, that it is almost total, that it is due to a decreased Vmax of transport, and that the rate of synthesis of the studied permeases is not modified during this inhibition. Furthermore, this substrate-mediated regulation also concerns other permeases, such as Can1, Lyp1, and Mup1. The mechanism of this activity regulation remains unknown to date. Study on a mutant Gap1 unable to enter the endocytotic pathway because it fails to be ubiquitylated [69] has confirmed that substrate inhibition affects the intrinsic activity of the permease and is not due to its internalization into endocytotic vesicles [78]. Furthermore, the group of C. Kaiser has reported that Gap1 is inactivated by an amino acid only if that amino acid has entered the cell via the Gap1 permease itself [79]. Additional studies integrating the data recently obtained on the predicted tertiary structure of amino acid permeases will be required to elucidate this regulatory mechanism, which probably acts on numerous other transporters in yeast and other organisms, but whose nature is not yet understood. 

Several authors reported in the late 60s that permeases undergo rapid turnover. It is true that when a protein synthesis inhibitor, such as cycloheximide, is added to a yeast culture, permease activities are rapidly lost. Yet, in a 1968 publication, M. Grenson, J.-M. Wiame, and students propose another interpretation of this observation: that cessation of protein synthesis causes an increase in the endogenous pool of amino acids, which in turn causes feedback inhibition of permeases. In agreement with this model, when a histidine mutant is deprived of histidine, the activity of several permeases decreases, probably because protein synthesis stops, but the activity of the histidine permease remains high [80]. More recently, it was shown that cycloheximide addition can inhibit certain permeases in another way: accumulation of endogenous amino acids activates TORC1, which then triggers permease ubiquitylation and endocytosis (see above) [81]. 

### 9.4. Regulation of Permease Activity According to Cell Ploidy

A study published in 1969 by C.F. Fox [82] showed that in an *E. coli* strain containing two copies of the *lac* operon, levels of catabolic enzymes are doubled, but the activity of the lactose permeases is only 10 to 40% higher than in a cell with only one lac operon. This observation suggested the existence of a factor that limits how much functional permease can be put in place, for instance, the available space in the membrane. This observation inspired a study that was published in 1970 by C. Hennaut, F. Hilger, and M. Grenson [83]. François Hilger was a student of J.-M. Wiame, who was trained in yeast genetics by M. Grenson. His contribution to this study consisted mainly in developing within the laboratory methods for isolating yeast triploids and tetraploids. The volume and surface area were then measured in n, 2n, 3n, and 4n yeast cells to deduce the surface-area-to-volume ratio, which was found, as expected, to decrease with increasing ploidy. Remarkably, C. Hennaut found the activities of the Can1, Lyp1, and Fui1 permeases to decrease also with increasing ploidy, proportionally to the change in the surface-area-to-volume ratio. This observation led the authors to consider that the space available for certain permeases in the plasma membrane is limiting, and that those permeases might even regulate their own production in order to avoid their excessive synthesis [83]. 

## 10. From Classical to Molecular Genetics in Permease Studies

### 10.1. Installing a New Laboratory on the Campus of the Plain in Ixelles

Shortly after J.-M. Wiame retired in the early 80s, M. Grenson left the IRC permanently to create a new laboratory on the ULB Campus of the Plain in Ixelles. She had two aims. The first was to continue her basic research on the diversity and regulation of amino acid permeases, notably by exploiting the gene cloning methods that researchers at the IRC were beginning to master. The second was to undertake applied research thanks to the support that was provided by several industrial research contracts, the most important of which was signed with a consortium of companies, called the SEPAM (*Syndicat d’Entreprises pour la Production d’Acides aminés*). The objective of this project was to isolate yeast mutants that were capable of synthesizing and excreting tryptophan in large quantities exploitable on an industrial scale. 

The SEPAM project relied on two lines of research that were previously conducted in the laboratory: the above-described study of amino acid excretion (cf. Section 8.6) and a study of tryptophan metabolism that was carried out in the first half of the 70s by Vang Nguyen Huu, under the supervision of J.-M. Wiame and M. Grenson. During his doctorate, V. Nguyen Huu had isolated tryptophan-overproducing mutants, mutants affected in certain tryptophan-catabolizing enzymes, and mutants combining these properties. These last displayed detectable tryptophan excretion, and the aim was to increase this production. The contract that was secured with the SEPAM in the early 80s aiming to extend this research enabled M. Grenson to hire Antonio Urrestarazu and Jacqueline Vu. A. Urrestarazu left the Basque Country of his native Spain and came to France to study philosophy and then biology. He then left France for a teaching job at the *Lycée français* of Istanbul, where he lived for several years. Attracted to molecular biology, in 1974 he enrolled as a student, in a master’s program (*licence spéciale*) that was organized by the Molecular Biology Department of the ULB, which had relocated from the *Rouge-Cloître* to the Rhode-Saint-Genèse Campus in the south of Brussels. A. Urrestarazu completed his master’s thesis and then undertook a doctorate in J.-M. Wiame’s laboratory. After two years of research in the USA, he returned to Belgium and joined the new team of M. Grenson. In 1982, J. Vu likewise did a master’s thesis (*licence spéciale*) in molecular biology in the laboratory of J.-M. Wiame, just before joining M. Grenson’s group. A. Urrestarazu, J. Vu, and a few others comprised the research team working on the industrial project devoted to tryptophan production. Towards the beginning of 1983, the SEPAM project team finally moved into laboratories that were located on the 7th floor of Building C of the Campus of the Plain in Ixelles. At the time, I was a young student living in student accommodations just facing Building C. I was intrigued to see the lights on both night and day on the top floor of that building. I was later to learn why: the research of the SEPAM team involved several fermenters, which were run night and day, and part of the staff was regularly required to monitor growth and perform analyses throughout the fermentation runs.


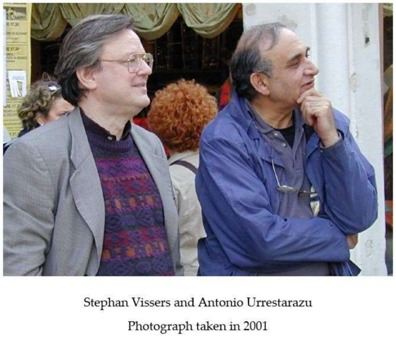


In 1983, shortly after the SEPAM team was assembled, additional space was made available to M. Grenson on the 6th floor of wing B of the same building. This space was used to house her basic research activities, which until then, were located at the IRC. Two other researchers from J.-M. Wiame’s team, Jean-Claude Jauniaux and Stephan Vissers, then decided to join her. After completing his studies at CERIA in 1966, Stephan Vissers was initially hired as a lab technician by J.-M. Wiame. He rapidly became highly competent in the study of the physiological regulation of arginine metabolism in yeast. Eager to learn more in relation to his research, S. Vissers took university courses, including those taught by Molecular Biology Department professors, such as R. Thomas, H. Chantrenne, and of course J.-M. Wiame. In 1980, he defended at the *Université de Paris-Sud* in Orsay a doctoral thesis on the regulation of the activity of arginine-metabolic enzymes in yeast cells according to their energy status. Although S. Vissers held J.-M. Wiame in high esteem, he also appreciated the human and scientific qualities of M. Grenson. He thus quite naturally decided to join her group just after J.-M. Wiame’s retirement. J.-C. Jauniaux, on the other hand, studied chemistry at the ULB and did his master’s thesis in 1974 in the group of Ilya Prigogine (laureate of the Nobel Prize in Chemistry, 1977). He then began a doctorate in the laboratory of J.-M. Wiame. Initially, in close collaboration with A. Urrestarazu, he studied the subcellular localization of the arginine-metabolic enzymes. Later, with M. Grenson, he carried out a genetic study of the organization of the *ARG5,6* gene, coding for two enzymes that were involved in arginine biosynthesis. J.-C. Jauniaux and S. Vissers also became involved, along with other IRC researchers, in introducing into the laboratory the basic genetic engineering techniques that were developed a few years earlier in the USA, which made it possible to clone and study yeast genes (see below). 

In addition to S. Vissers and J.-C. Jauniaux, M. Grenson’s basic research team included F. Muyldermans and Marc De Baerdemaeker, the latter also a technician hired by the IRC and who had worked closely with J.-C. Jauniaux. Each year the group was enriched with one or several students, including Micheline Vandenbol, who was a physicist who had completed an additional master’s degree (*licence spéciale*) in molecular biology and who joined the laboratory in early 1984 (first for her master’s thesis, then for a doctorate). It is worth mentioning that the group’s trio of senior researchers, S. Vissers, J.-C. Jauniaux, and A. Urrestarazu, had already forged strong friendships at the IRC. In late 1984, another ex-member of J.-M. Wiame’s research group, Kathleen Broman, joined the team just after returning to Belgium from a post-doc in the USA. 

My first contact with M. Grenson’s laboratory dates back to 1985, when I spent a few days there as an intern in the framework of my biology studies at the ULB. The aim of the experiment that we tried to carry out, under the supervision of J.-C. Jauniaux and M. De Baerdemaeker, was to test a new method for transforming yeast cells, which was just developed by Ito et al. [84]. The method was based on treating the cells with lithium, and was an alternative to the much more laborious transformation method based on preparing spheroplasts. Our experiment totally failed, but that did not prevent me from joining the laboratory in October of 1986, for my doctorate. J.-C. Jauniaux, thanks to his availability and convincing arguments, played a key role in this decision. 

The installation of her new laboratory on the campus in Ixelles, together with the reinforcements to her team, coincided with the beginning of a new, exalting period in M. Grenson’s research career. Among other achievements, this period was marked by successful cloning of several permease genes (see below). It was also marked, sadly, by the gradual decline of M. Grenson’s health due to hepatitis C, a condition likely contracted through blood transfusions necessitated by the consequences of her automobile accident in September 1970. Furthermore, there was increasing pressure on the laboratory’s SEPAM group to meet certain tryptophan production objectives, and this further increased her growing level of fatigue. In 1987, during the party following J.-C. Jauniaux’s thesis defense (he defended his thesis belatedly), M. Grenson suffered from a serious attack and had to be urgently hospitalized. This left the whole team thunderstruck, and also J.-M. Wiame, who was present at the party. 

In the following pages, I summarize the scientific achievements of M. Grenson’s group during the 80s and 90s up to the time of her retirement.

### 10.2. Isolation of Mutations in the PMA1 Gene, in Collaboration with A. Goffeau

Before coordinating the European yeast genome sequencing project, André Goffeau, Professor at the Catholic University of Louvain (UCL), focused principally on the biochemical study of the proton pump that is present in the yeast plasma membrane. This membrane ATPase plays a key metabolic role by establishing an H^+^ gradient across the plasma membrane. A. Goffeau and M. Grenson knew and liked each other well. In the early 80s, they began a collaboration aiming to isolate mutants altered in the *PMA1* gene, encoding the ATPase, with a view to cloning this gene. M. Grenson isolated some 400 mutants resistant to the toxic compound Dio-9, described as an inhibitor of the *in vitro* activity of the proton pump. In-depth analysis of four of these mutants revealed that they all carried semi-dominant mutations affecting the same genetic locus. Biochemical experiments that were performed by Stanislaw Ulaszewski in A. Goffeau’s laboratory then showed that, in the mutant strains, the proton pump showed partial resistance to several of its well-known inhibitors (such as vanadate and miconazole), an increased Km for ATP, and other anomalies. He concluded that these mutants most likely carried mutations in the *PMA1* gene [85]. One of the mutations was then mapped to a site on chromosome 7, near the *LEU1* gene. A DNA fragment complementing a *leu1* mutation was then isolated, and it turned out to carry the *PMA1* gene. The report on this work, published in 1987 by A. Goffeau and coworkers [86], appeared shortly after the paper published in *Nature* by Ramon Serrano, Morten Kielland-Brandt, and Gerry Fink, describing the cloning and sequencing of the *PMA1* gene [87]. A. Goffeau and M. Grenson also worked on another topic: the transport of K^+^ ions. To identify genes coding for K^+^ transporters, M. Grenson again developed a genetic approach, based on the isolation of rubidium-resistant mutants. The isolated strains were not exploited in cloning experiments, however. 

### 10.3. Molecular Study of the Diversity and Regulation of Amino Acid Permeases

The first studies that were describing the application of the new genetic engineering methods to yeast appeared in the late 70s. Several researchers at the IRC promptly applied these methods and proceeded to clone yeast genes of interest, particularly those that were involved in arginine metabolism and amino acid transport. These scientists were able to count on the experience that was gained in this area by M. Crabeel who, after her thesis in M. Grenson’s group, had joined the laboratory of Nicolas Glandsdorff in the part of the CERIA that was associated with the VUB (Flemish *Vrije Universiteit Brussel*). M. Crabeel, after an internship in 1975 in the laboratory of Werner Maas at the New York University Medical School, was indeed the first in the Anderlecht laboratory to exploit the rare restriction enzymes that were available at the time. She thus cloned, already in 1976, the *E. coli* operon involved in arginine biosynthesis [88]. In 1979, J. Broach, J. Strathern, and J. B. Hicks of the Cold Spring Harbor Laboratory reported the use of a vector that could be used to clone yeast fragments carrying a gene of interest by simple complementation of a defect in that gene, which was present in a mutant yeast strain. As mentioned above, these researchers demonstrated the efficacy of this new cloning method by isolating the *CAN1* gene, encoding the arginine permease, which was identified more than twenty years earlier by M. Grenson [43].


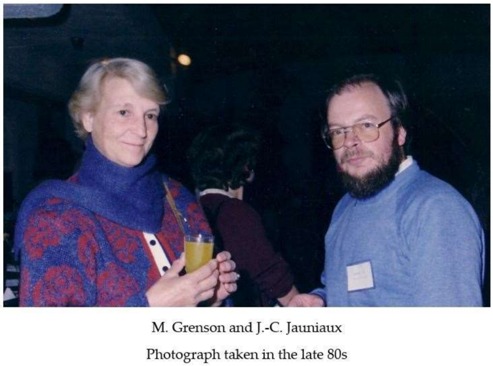


J.-C. Jauniaux and S. Vissers were among the IRC researchers who, with E. Dubois and Francine Messenguy, invested time and effort in developing yeast gene cloning methods. First results obtained from implementing these methods were published in 1982, notably the cloning of the *CAR1* gene (coding for arginase) [89] by complementation of the defect of the AG1 mutant that was isolated twenty years earlier by M. Grenson. S. Vissers remembers the day when, for the first time, he and J.-C. Jauniaux used a UV lamp to visualize DNA that was extracted from yeast in an ethidium-bromide-soaked agarose gel, after its migration through the gel. Having marveled at this result for long minutes, they returned home with what looked like sunburn on their faces. When the two colleagues later joined M. Grenson’s group in 1983, they first continued projects that begun at the IRC, aiming to clone the *GAP1* and *NPR1* genes. They failed at their many attempts to clone the *GAP1* gene by simple complementation of a *gap1* mutation, probably because of the insufficient quality of the genomic library that was used at the time. J.-C. Jauniaux then imagined an alternative cloning strategy. At the IRC, he had shown by Southern blotting that the promoter regions of certain genes, including the arginase gene, which contained an inserted Ty1-type retrotransposon [89]. Thanks to his knowledge of this type of mobile genetic element, he developed a transposon-tagging approach to clone the *GAP1* gene. This involved introducing into a *ura3 car1* yeast strain a plasmid bearing the *CAR1* gene and a Ty1 element into which a *URA3* gene had been inserted. These yeasts were then grown in order to identify spontaneous *gap1* mutants with a *URA3* genetically linked to a *gap1* mutation. In 1987, after several years of efforts, the *GAP1* gene was finally cloned by means of this approach [90]. Expression of the *GAP1* gene was then studied by northern blotting, in the wild type and in mutant strains showing deregulated Gap1 activity. It took several more years to establish the complete sequence of the gene, and all of these results were finally published in 1990, in a paper that was authored by J.-C. Jauniaux and M. Grenson that to date is cited more than 260 times [91]. 

The first permease gene to be cloned successfully by M. Grenson’s group was *PUT4*, encoding the proline permease [92]. Sequencing of this gene, isolated by complementation of the defects preventing utilization of proline and GABA by a *gap1 put4 uga4* mutant (see 10.4), was completed in 1988 [93]. Bioinformatic analyses—then in their infancy—revealed considerable primary sequence similarities between Can1, Hip1, Put4, and Gap1 (similarities between the first two permeases were evidenced already in 1986). These permeases thus defined a family of conserved proteins, about twenty of which exist in yeast.

M. Vandenbol, who participated actively in sequencing the *PUT4* gene [93], also cloned (by complementation) the *NPR1* gene encoding the positive regulator of the activity of several ammonium-inactivated permeases [94]. I remember the sudden excitement in the group when the screen of the computer on which M. Vandenbol was analyzing her results revealed a high percentage of identity between the Npr1 protein and protein kinases that are involved in the cell cycle. The hypothesis that naturally emerged from this result was that Npr1 activates permeases by phosphorylating them [95]. As indicated above, later studies showed that a variety of mechanisms enable Npr1 to regulate permease activities through phosphorylation (cf. 9.1 and 9.2). 

### 10.4. Genetic and Molecular Study of the UGA Genes, Involved in GABA Uptake and Catabolism

4-Aminobutyric acid (GABA) is an amino acid that is well known as a neurotransmitter in mammals. It can also be used as a nitrogen source by yeast, and the work by F. Ramos and collaborators showed in 1985 that the two enzymes involved in its utilization, a transaminase (Uga1) and a dehydrogenase (Uga2), are induced when GABA is present in the external medium [96]. In collaboration with M. Grenson, *uga1* and *uga2* mutants had been isolated. A third class of mutants, *uga3*, turned out to be greatly impaired in the induction of these two enzymes, and this suggested the existence of a positive regulator of this induction. M. Grenson and her team later studied GABA transport. They showed that GABA uptake involves Gap1 and two additional permeases, one of which is the proline permease Put4, which was previously described by Marjorie Brandriss and coworkers [97]. The involvement of Put4 was deduced from the observation that GABA uptake into a *gap1* mutant grown on urea medium is competitively inhibited by proline. A *put4* mutant was then isolated as described by the group of M. Brandriss [97]. In the *gap1 put4* double mutant, GABA uptake is low, but it increases over time upon the addition of GABA to the medium. This indicated the existence of a third GABA permease, which was inducible by its substrate. A mutant that was deficient in this permease, called Uga4, was then isolated from the *gap1 put4* strain by seeking clones unable to use GABA as a nitrogen source. This complete study of GABA transport, which illustrates well the methodology established by M. Grenson for dissecting on a genetic basis the uptake routes of a given amino acid, was published in 1987 [98] in an Indian journal which had just appeared, and which M. Grenson wanted to support, but whose distribution was unfortunately limited and which has disappeared since.

When I approached M. Grenson during the summer of 1986 to ask about undertaking a doctorate in her laboratory, she presented to me the first results, which were just obtained by S. Vissers, of the molecular study of the *UGA* genes. As starting point of my thesis, she proposed that I clone the *UGA3* gene. This was thus my first research subject, which led to the identification of a transcriptional activator containing a zinc finger of the same type as is present in the Gal4 factor [99]. Additional experiments that were carried out together with S. Vissers and F. Muyldermans enabled us to dissect in detail the GABA-sensitive regulon. We isolated a constitutively active, dominant mutant form of the Uga3 factor, and this suggested that this factor is directly activated by GABA [99,100]. We also identified two additional regulatory genes, *UGA35* and *UGA43*, which are involved in controlling the *UGA* genes. Unlike *UGA3*, these two genes later turned out to participate in the transcriptional control of additional inducible genes, such as those that are involved in the utilization of urea [101]. They thus appeared as more pleiotropic regulatory factors. David Coornaert, M. Grenson’s last doctoral student, joined the lab in early 1989. He cloned and sequenced the *UGA35* and *UGA43* genes, showing that they coded for proteins (Uga35/Dal81 and Uga43/Dal80) having each a zinc-finger-type DNA-binding domain [102,103]. Finally, it was the whole set of structural and regulatory genes that was cloned and sequenced in the laboratory in the early nineties [99,102,103,104,105], at a time when research into the transcriptional control of gene expression was booming. 

## 11. Retirement and Last Links with the Laboratory

In 1990, M. Grenson reached the official age of retirement. This is a period in which great uncertainty reigned regarding the future of the laboratory. None of the researchers in the team belonged to the academic staff, and holding a tenured position was a prerequisite to becoming the head of the laboratory. This situation sparked the greed of some of our space-hungry colleagues: I remember them visiting our working areas while we were conducting our experiments, even taking measurements, anticipating that they would soon be able occupy them. Of course this aroused the anguish of the whole team. M. Grenson then spoke of her concerns to Professor Gisèle Van de Vyver, who was a biologist that specialized in freshwater sponges and who was then Dean of the Science Faculty. At the end of their discussion, M. Grenson proposed to her that she be her successor at the head of the laboratory. This would enable her to exploit the skills of M. Grenson’s team to clone genes involved in the developmental process in sponges, while ensuring that the research on yeast could continue. G. Van de Vyver, after thinking it over, accepted the proposal and thus became the official head of the laboratory. 

The arrival of G. Van de Vyver at the head of the laboratory was preceded by other major changes to the team. Already in early 1987, the SEPAM consortium decided suddenly to stop funding the tryptophan production project. This forced M. Grenson to lay off all of the members of the team on the 7^th^ floor, with the exception of A. Urrestarazu. This was a very distressing situation, given the huge investment that was made by the group to meet the industrial demands. In 1989, F. Muyldermans took a well-deserved early retirement, and the technician position she had held became vacant. The job was offered to Catherine Jauniaux (sister of J.-C. Jauniaux), who had been trained as a lab technician at the CERIA and had been hired first as a student worker and then (in early 1989) as a lab technician. During my doctorate, it was my pleasure to train C. Jauniaux in several genetic engineering methods applied to yeast. This created close ties between us, which even now constitute the basis of our working relationship. C. Jauniaux is currently the principal technician of my laboratory. 

The end of the 80s was also marked by the beginning of the laboratory’s involvement in the yeast genome sequencing project. This came about quite naturally due to the well-established contacts that M. Grenson and J.-C. Jauniaux had with A. Goffeau, the initiator of the European sequencing effort. From 1989, the plan was to actively participate in obtaining the complete sequence of chromosome III. The European effort was a great success and chromosome III became the first eukaryotic chromosome to be sequenced [106]. J.-C. Jauniaux was the linchpin of this project in the lab. However, in 1992, he left the group to accept, as did also his wife, a research position at the German Cancer Research Center (DKFZ), where their colleague Jean Rommelaere had just been hired to create a laboratory devoted to the study of parvoviruses. After the departure of J.-C. Jauniaux, between entre 1992 and 1996, A. Urrestarazu shouldered the task of supervising the sequencing efforts that contributed sequence information regarding other yeast chromosomes. 

Just after her retirement, M. Grenson continued to visit the laboratory regularly. This offered the opportunity to discuss the evolution of our models aiming to explain the transcriptional regulation of the *UGA* genes. These visits, however, became less and less frequent as her health declined. In 1991, I wrote and presented my doctoral thesis, and during that period M. Grenson gave me more than kind support. After my military service, I returned to the lab in September 1992 to continue the study of the transcriptional regulation of the *UGA* genes, notably with a young graduate student, Driss Talibi, who had meanwhile joined the team. In January 1993, two young chemistry students, Anna Maria Marini and Jean-Yves Springael, joined the laboratory for their masters’ theses, and each of them continued with a doctorate. Rather than study more in depth the regulation of the *UGA* genes, I suggested to A.M. Marini that she clone the *MEP1* gene, encoding one of the ammonium transporters [58]. The subject proposed to J.-Y. Springael was to clone the *NPI1* gene required for the inactivation of ammonium permeases by ammonium [58,68]. In 1994, just after our publication on the molecular characterization of the very first ammonium transporter (Mep1) [58], I paid a visit to M. Grenson in her home in Hoeilaart. At that time, she practically never came to the lab, and I was in the habit of visiting her to see how she was doing and to give her an update on the research in progress. On that day M. Grenson had just received a phone call from her doctor, informing her that she had developed cancer of the liver, the consequence of having hepatitis C, which sadly is not an uncommon complication. A few months later, my wife and I visited her to introduce her to our newborn daughter Ségolène. This visit delighted Marcelle, for although she never had any children, she adored them. On that visit, we noticed a clear decline in her general health. In the months that followed, she was hospitalized several times, at decreasing intervals. M. Grenson died in Ixelles Hospital in February of 1996, in grim circumstances, but surrounded by her companion Raymond Quackels and by many of her friends. 


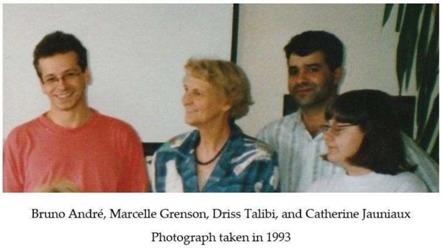


Marcelle Grenson devoted herself wholeheartedly to scientific research. Her students and collaborators appreciated her intellectual rigor and her outstanding logic. Some of them remember that to warn them against logical fallacy, she liked to tell the story of a flea trainer who had taught his fleas to jump at his command. When he observed that when he removed their back legs they no longer responded, he logically but erroneously concluded that fleas hear through their legs. M. Grenson was naturally pleasant and smiling, and also readily available, but she could also be severe. She often practiced irony, and her humor, always subtle, could be biting. She was generous towards and attentive to each member of her team. Although this tribute is focused on her scientific achievements, M. Grenson had many other centers of interest. She read a lot and even created a small library in the laboratory where she left non-scientific books that she invited us to read. She was also interested in art. She painted pictures with watercolors and pastels, some of which are displayed today in the laboratory. She loved classical music with passion, but also liked other musical styles: once when on one of my visits she was showing me her rich collection of records, I was surprised to see that she owned singles with progressive rock. This brought us even closer. She explored philosophy and practiced philanthropy, and she defended human rights through her involvement with Amnesty International. One day she received a surprise visit from a Chilean ex-prisoner whose release she had helped to obtain. Her house in Hoeilaart was surrounded by a large garden where she grew flowers and which she loved to show us when we visited her during the growing season. Although she has left us, we very often refer to her work during our discussions in the lab. Her scientific opus has been and remains a reference base for the studies carried out in my lab, as for the work of other research teams exploring membrane transport. 

## Appendix A

Strain F35, which was sent by M. Grenson to G. Fink in September of 1970, has an *apf* mutation which M. Grenson and C. Hennaut later described [37] and which is allelic with the *aap* mutation described in 1965 by Surdin et al. [35]. This gift of the *aap* strain resulted from a visit of J.-M. Wiame and M. Grenson to the USA and the laboratory of G. Fink (then Assistant Professor at Cornell University, Ithaca) and from a letter sent on 19 August, 1970 by G. Fink to M. Grenson (we have found this letter). Strain F35 bears a mutation in the *APF1/SHR3* gene and therefore shows strongly reduced amino acid transport. The strain subcultured by Carlos Gimeno in G. Fink’s laboratory was a diploid, since otherwise no pseudohyphae would have been observed. Presumably the strain sent by M. Grenson diploidized during successive subculturing in G. Fink’s laboratory, at a time when cryopreservation of yeasts was not frequent. This is what G. Fink’s technician Cora Styles explains in her description of the experiments having led to the rediscovery of pseudohyphal growth thanks to the *S. cerevisiae* strain Σ1278b (see www.yeastgenome.org).

## Appendix B

It was totally justified to address this question, because the important work of Roseman and Kaback begun in 1963 on the PEP-phosphotransferase system had shown that glucose is phosphorylated upon its transport. G. Cohen and J. Monod were to show, however, that the same does not apply to β-galactosides and amino acids, which accumulate in *E. coli* in unmodified form. M. Grenson and students showed that amino acids likewise accumulate as such in yeast.

## Appendix C

Note that J. Gits used l-ethionine at low dosage to isolate these mutants. If the concentration used is too high or if a mixture of l- and d-ethionine is used, the mutants isolated are mostly strains deficient in several permease activities (*apf*-type mutants), because the uptake of ethionine at high concentration can be effected by several permeases. J. Gits thus isolated mutants resistant to a low dose of l-ethionine and then ignored the mutants displaying resistance to other toxic amino acid analogs such as canavanine and thiosine.

## Appendix D

François Lacroute clearly explains the context of his work in the first pages of his thesis (our laboratory has the copy he gave to M. Grenson). As it seemed that the nucleic acid affected by the “petite” cytoplasmic mutations might be RNA (this was the mainstream hypothesis at the time, because of the presence of RNA in the cytoplasm), F. Lacroute tested the influence of uracil and thymine analogs on the appearance of ρ^-^ mutants from a diploid strain. He thus observed that “petite” mutants arose in the presence of 5-fluorouracil.

## Appendix E

It was in the late 60s that M. Grenson isolated a first set of mutants in which Gap1 is active despite the presence of ammonium. One approach was to start with an arginine auxotroph additionally deficient in the arginine permease, and thus unable to grow on arginine-containing ammonium medium because Gap1 is inhibited. Mutants were isolated for their ability to grow on this medium. Another approach was to isolate, from an *arg5,6* arginine auxotroph, mutants capable of using citrulline (a specific Gap1 substrate) as a source of arginine, despite the presence of ammonium in the medium. A mutant obtained by the first approach, showing a reduced growth rate on ammonium medium, was studied by Claire Hou during her doctorate. She found this mutant to be altered in the main enzyme involved in ammonium assimilation, the NADPH-dependent glutamate dehydrogenase encoded by the *GDH1* gene [107]. To further demonstrate that the mutant (called *gdhA*) carried a mutation in the structural gene of the dehydrogenase, M. Grenson later showed, in collaboration with her Strasbourg colleagues Robert Drillien and Michel Aigle, that other mutations at the same locus can alter the K_m_ of the enzyme for one or another of its substrates. They can also give rise to intragenic complementation or negative dominance, two phenomena expected of this enzyme, which was known to form oligomers [108]. Later, a fruitful collaboration between E. Dubois, M. Grenson, and J.-M. Wiame, initiated in 1972, showed that nitrogen catabolic repression (NCR), known to affect the synthesis of certain enzymes involved in nitrogen catabolism, was also relieved in the *gdhA* mutant [109]. On the basis of this observation, E. Dubois et M. Grenson obtained and analyzed additional mutants affecting NCR of certain catabolic enzymes [110]. Among these were the *gdhCR/ure2* mutations, also obtained by F. Lacroute’s group in Gif-sur-Yvette, and those affecting the glutamine synthetase gene (*GLN1*), whose contribution to NCR had been reported by Boris Magasanik’s group. In ammonia-grown *gdhCR/ure2* mutants, Gap1 activity remains low on ammonium, even though the NCR of certain enzymes is totally relieved. It was concluded that the inhibitory mechanism affecting Gap1 activity is still active in these mutants. This situation differs from that of the *gdhA* mutant, where both repression of Gap1 synthesis and inhibition of its activity are impaired [107]. M. Grenson thus decided to repeat the mutagenesis she had performed on the *arg5,6* mutant, but to include an additional NCR-suppressing *gln1(ts)* mutation. Genetic analysis of mutants selected for their ability to use citrulline as a nitrogen source in the presence of ammonium revealed that they contained different types of mutations, including *gdhA*, *mep-1*, other mutations initially called *mut* and later renamed *npi*, and semi-dominant *gap1(pgr)* mutations located in the *GAP1* gene. These last were studied and mapped by Bernadette Acheroy, a graduate student who joined M. Grenson’s team in 1978 [111]. In double mutants combining a *gln1(ts)* or a *gdhCR* mutation with an *npi* or a *gap1(pgr)* mutation, the Gap1 permease is highly active on ammonium medium, because both repression of its synthesis and inhibition of its activity are impaired. This is clearly illustrated in a paper [66] exploiting the graphic representation of differential rates of synthesis devised by J. Monod and coworkers.

As indicated above, mutants where Gap1 remains active on ammonium medium were isolated from an *arg5,6* mutant for their capacity to use citrulline as source of arginine in the presence of ammonium. These mutants, initially called UT1 and later renamed *uta*, were also studied by C. Hou during her doctorate. On the basis of her results, one can imagine that the *uta* mutation is—or is equivalent to—one of the *mut/npi* mutations that were isolated later.

The study of the *NPR1* locus was initially conducted independently, or almost so, of the investigation of the ammonium regulation. Mutations in this gene were isolated in at least two different ways. One approach started with a *dur1,2* strain deficient in the enzymes enabling yeast cells to utilize urea as a nitrogen source. This strain is inhibited by the presence of allantoin, and Rosette Loriau, a technician in the laboratory, isolated mutations that suppressed this toxicity. She showed that some of the suppressing mutations slowed growth on ammonium medium and largely relieved the NCR exerted on certain enzymes in the presence of ammonium [75]. Around the same time, M. Grenson, R. Loriau, and E. Dubois were also studying the ammonium permeases. It appeared that the *npr1* mutation greatly reduces the activity of these permeases, and also that of Gap1 and other ammonium-sensitive permeases [57]. In other words, Npr1 appeared as a positive factor in the regulation of the activity of several permeases whose activity is inhibited by ammonium. In agreement with this model, when B. Acheroy isolated *gap* mutants by selecting clones resistant to d-amino acids, she also obtained *npr1* mutant strains [111]. A key step in the effort, begun in the 60s, to elucidate the mechanisms underlying the negative regulation of Gap1, was the discovery of the link between Npr1 and Gap1 inhibition by ammonium. This link was clearly established when strains containing an *npr1* mutation, but having recovered high Gap1 activity, were found to contain an additional *mut/npi* or *gap1(pgr)* mutation, just like the *arg5,6 gln1(ts)* cells having become resistant to inhibition by ammonium (mentioned above in the present endnote) [111]. These findings constitute the basis of the experiments published by M. Grenson in 1983, leading to the integrated model of the regulation of Gap1 synthesis and activity by ammonium, described in the main text of this tribute [66,112].
